# Gene products of chromosome 11q and their association with *CCND1 *gene amplification and tamoxifen resistance in premenopausal breast cancer

**DOI:** 10.1186/bcr2150

**Published:** 2008-09-29

**Authors:** Katja Lundgren, Karolina Holm, Bo Nordenskjöld, Åke Borg, Göran Landberg

**Affiliations:** 1 Center for Molecular Pathology, Department of Laboratory Medicine, Lund University, Malmö University Hospital, Malmö, SE-205 02, Sweden; 2 Department of Clinical Sciences, Division of Oncology, Lund University, Lund, SE-221 85, Sweden; 3 Department of Oncology, Borås Hospital, Borås, SE-501 82, Sweden; 4 Division of Oncology, Faculty of Health Sciences, Linköping University, Linköping, SE-581 85, Sweden

## Abstract

**Introduction:**

The amplification event occurring at chromosome locus 11q13, reported in several different cancers, includes a number of potential oncogenes. We have previously reported amplification of one such oncogene, namely *CCND1*, to be correlated with an adverse effect of tamoxifen in premenopausal breast cancer patients. Over-expression of cyclin D_1 _protein, however, confers tamoxifen resistance but not a tamoxifen-induced adverse effect. Potentially, co-amplification of an additional 11q13 gene, with a resulting protein over-expression, is required to cause an agonistic effect. Moreover, during 11q13 amplification a deletion of the distal 11q region has been described. In order to assess the potential impact of the deletion we examined a selected marker for this event.

**Method:**

Array comparative genomic hybridization analysis was employed to identify and confirm changes in the gene expression of a number of different genes mapping to the 11q chromosomal region, associated with *CCND1 *amplification. The subsequent protein expression of these candidate genes was then examined in a clinical material of 500 primary breast cancers from premenopausal patients who were randomly assigned to either tamoxifen or no adjuvant treatment. The protein expression was also compared with gene expression data in a subset of 56 breast cancer samples.

**Results:**

Cortactin and FADD (Fas-associated death domain) over-expression was linked to *CCND1 *amplification, determined by fluorescence *in situ *hybridization, but was not associated with a diminished effect of tamoxifen. However, deletion of distal chromosome 11q, defined as downregulation of the marker Chk1 (checkpoint kinase 1), was associated with an impaired tamoxifen response, and interestingly with low proliferative breast cancer of low grade. For Pak1 (p21-activated kinase 1) and cyclin D_1 _the protein expression corresponded to the gene expression data.

**Conclusions:**

The results indicate that many 11q13 associated gene products are over-expressed in conjunction with cyclin D_1 _but not linked to an agonistic effect of tamoxifen. Finally, the deletion of distal 11q, linked to 11q13 amplification, might be an important event affecting breast cancer outcome and tamoxifen response.

## Introduction

Gene amplification is a well defined cause of oncogene activation during tumor development, and some genomic regions are recognized to be more frequently amplified than others [1].

Amplification of chromosome locus 11q13 occurs at high frequencies in certain human cancers, including lung, bladder, breast and ovarian carcinomas, as well as in head and neck squamous cell carcinomas (HNSCCs) [2-6]. Approximately 15% of primary breast cancers are affected by this specific amplification, which is associated with poor prognosis [7-10]. Four distinct core regions or amplicons within the 11q13 locus have been identified, and these can be amplified independently or concurrently in various combinations [7,11]. A number of oncogenes or potential cancer-related genes have been mapped to the 11q13 chromosomal region. The *CCND1 *and *CTTN *oncogenes have been putatively proposed as candidate genes for the emergence and maintenance of this amplification event in breast cancer [3,7]. These genes map to two different amplification cores, located within 0.8 megabases of each other at 11q13.3 [2,11], and their co-amplification has been reported in breast cancer [1,2,12]. The core region comprising *CCND1 *is the most frequently amplified and is involved in two-thirds of the amplifications. The *CCND1 *gene is the most extensively studied gene of the 11q13 amplification region, and encodes the cell cycle regulatory protein cyclin D_1_, which is important both for development of mammary tissue and in mammary carcinogenesis [2].

In breast cancer, amplification and over-expression of cyclin D_1 _has been associated with worse prognosis [13,14], but high expression of cyclin D_1_, in contrast, has also been associated with better prognosis [15,16]. Breast cancer patients exhibiting estrogen receptor (ER)-α expression, with concurrent over-expression of cyclin D_1_, have been reported not to benefit from treatment with the selective estrogen receptor modulator tamoxifen, which is in contrast to the evident response in ER-α-positive breast cancers with moderate and low cyclin D_1 _expression [15]. Jirstrom and coworkers reported that *CCND1 *amplification was associated with a potential agonistic effect of tamoxifen in ER-α-positive premenopausal breast cancer patients, even when not accompanied by protein over-expression [17].

*INT2*, *FADD*, *PAK1*, and *EMSY *are other candidate genes reported to be included in the 11q13 amplicon [7] and thier amplification or protein over-expression has been associated with a poor prognosis in various cancers [1,2,8,9,18-20].

It has been reported that in HNSCC amplification of 11q13 involves a loss of distal chromosome 11q (from 11q14.2 to 11qter) through a breakage-fusion-bridge cycle mechanism [21]. In this process, genes with important roles in, for instance, the DNA damage response are lost in the deletion step preceding the amplification. Frequent allelic deletions at chromosome 11q24–q25 have been reported in both breast and ovarian cancer and have been associated with a worse clinical outcome [22,23].

A potential adverse effect of tamoxifen in *CCND1 *amplified breast cancers is indeed intriguing. As noted, over-expression of cyclin D_1 _protein has been linked to lack of tamoxifen response but not to a direct agonist effect. Hypothetically, another gene co-amplified with *CCND1 *might be responsible for the agonistic effect of tamoxifen. Furthermore, genes deleted in the 11q13 amplification event might also affect breast cancer outcome and treatment response.

In order to elucidate the importance of 11q-associated genes with regard to tamoxifen response and *CCND1 *amplification, we identified previously described candidate genes at the 11q chromosomal region for further investigation, using array comparative genomic hybridization (CGH) analysis of breast cancer samples. Protein expression levels of the various cancer-related gene products were then analyzed in a tissue microarray from a randomized trial of premenopausal breast cancer patients receiving 2 years of adjuvant tamoxifen treatment or no adjuvant treatment. By comparing treated and untreated patients, we were able to delineate the response to tamoxifen irrespective of prognostic features, and a potential agonistic or diminished effect of tamoxifen could be identified. Furthermore, the tumor material allowed us to study prognostic features, relations with different clinicopathological parameters, and associations with *CCND1 *amplification, in different subgroups defined by the expression of the 11q13 and distal 11q gene products. The results indicate that many 11q13-associated gene products are over-expressed in conjunction with cyclin D_1 _but are not linked to an agonistic effect of tamoxifen. Conversely, deletion of the distal end of chromosome 11q, defined as downregulation of the marker Chk1 (checkpoint kinase 1), was associated with an impaired tamoxifen response, and with low proliferative breast cancer of low grade.

## Materials and methods

### Comparative genomic hybridization

Array CGH was performed essentially as was previously described [24]. Raw data and normalized data are available through National Center for Biotechnology Information Gene Expression Omnibus [GEO: GSE12759].

### Patient materials

Between 1986 and 1991, a total of 564 premenopausal breast cancer patients with invasive stage II disease were enrolled in a Swedish trial (SBII:2a), in which they were randomly assigned to 2 years of adjuvant tamoxifen (*n* = 276) or no adjuvant treatment (control; *n* = 288). The aim of the original study was to compare 2 years of tamoxifen treatment (20 or 40 mg/day) versus no adjuvant treatment. Patients were included irrespective of hormone receptor status. All patients were followed up for recurrence-free survival (RFS) and overall survival. Recurrence was defined as local, regional, or distant recurrence, and breast cancer-specific death, whereas contralateral breast cancer was excluded. Surgery was modified radical mastectomy or breast conserving surgery, followed by radiotherapy and, in a few cases, adjuvant polychemotherapy (in <2% of cases). The time of surgery defined time point zero in this study. The patient median follow-up time without breast cancer event was 13.9 years. Detailed description of the SBII:2a study design can be further viewed in a previous report [25]. Informed consent was obtained from the patients for the initial randomized study, and the ethics committees at Lund and Linköping Universities that approved the study did not require additional consent for the present study.

### Tissue specimens and immunohistochemistry

Formalin-fixed and paraffin-embedded tumor material was available from 500 of the 564 patients in the trial. Areas representative of invasive cancer were selected and assembled in a tissue microarray. Two 0.6 mm tissue cores from each donor block were placed in recipient paraffin blocks by using an automated tissue arrayer (Beecher Instruments Microarray Technology, Woodland, MD, USA). Sections (4 μm) from this block were mounted onto slides before they were deparaffinized, rehydrated, and microwave treated in target retrieval solution pH 9.9 (Dako, Glostrup, Denmark), before undergoing processing in an automated immunostainer (Techmate 500; Dako, Copenhagen, Denmark), using the Envision software (Dako, Glostrup, Denmark). The antibodies used were mouse monoclonal anti-human cortactin (1:50, clone 30; BD Biosciences, Erembodegem, Belgium), mouse monoclonal anti-human FADD (Fas-associated death domain; 1:50, clone A66-2; BD Biosciences), and mouse monoclonal anti-human Chk1 (1:100, clone 2G1D5; Cell Signaling, Danvers, MA, USA). For Chk1, both nuclear staining intensity and fraction positive nuclei were evaluated. The variable designating fraction Chk1 positive nuclei was best suited for describing the appearance of Chk1. Staining was evaluated by two independent observers (one pathologist), in order to obtain a result as correct and representative as possible. Conflicting observations were low (<5%) for all three evaluations made. All immunohistochemical (IHC) evaluations were performed without knowledge of tumor characteristics. In cases of no evaluation, cores were either nonrepresentative (contained no invasive tumor cells) or missing.

Data for expression of ER-α (a combination of IHC and enzyme immunoassay) were available from a previous study, in which ER-α positivity was assessed according to the Swedish clinically established cutoff of 10% positively stained nuclei [25].

Data for expression of Pak1 (p21-activated kinase 1) [26], cyclin D_1_, and *CCND1 *gene amplification status (done by fluorescence *in situ *hybridization [FISH] analysis) [17] were also available. When the ratio of intensity of the *CCND1 *probe to the centromere probe was greater than 1 in at least 20% of the tumor cells, the gene was considered to be amplified. In addition, expression of the proliferation marker Ki67 had also been evaluated in a previous study [27].

### Chromogenic *in situ *hybridization

Chromogenic *in situ *hybridization (CISH) was performed in accordance with the Zymed SPoT-Light Cyclin D1 Probe protocol, which is well suited to CISH [28], using the SPoT-Light Cyclin D1 Amplification Probe (Zymed laboratories, Invitrogen immunodetection; San Francisco, CA, USA). Pretreatment procedures included heat pretreatment and enzyme digestion to optimize the CISH performance.

### Cell lines, Western blot, and immunocytochemistry analyses

The human breast cancer cell lines CAMA-1, MCF-7, T-47D, MDA-MB-468, and MDA-MB-231 (ATCC, Manassas, VA, USA) were used to verify the reactivity of the cortactin, FADD, and Chk1 antibodies, by Western blot and immunocytochemistry (ICC). For detailed culturing conditions, and ICC and Western blot analyses, we refer to the methods described by Holm and coworkers [26]. MCF-7 cells were grown in Improved MEM (Minimum essential media) zinc option (Gibco, Grand Island, NY, USA) supplemented with 5% fetal bovine serum, and all culture media were supplemented with 1% penicillin/streptomycin. For ICC, an array of these cell lines was constructed and stained with the cortactin, FADD, and Chk1 antibodies separately.

For Western blot, 20 μg of each protein sample was resolved on SDS-polyacrylamide gels and transferred to Hybond ECL nitrocellulose membranes (Amersham Pharmacia Biotech, Amersham, Buckinghamshire, UK). Membranes were incubated with cortactin (1:1,000), FADD (1:250), Chk1 (1:1000), and polyclonal goat anti-human β-actin (1:500; Santa Cruz, Biotechnology, Santa Cruz, CA, USA) antibodies for 2 hours, followed by incubation with secondary horseradish peroxidase-conjugated anti-mouse (Amersham Life Science, Aylesbury, UK) and anti-goat antibodies (Sigma, Gothenburg, Sweden) for 1 hour. Membrane-bound antibody was detected by using the ECL^+ ^system (Amersham Life Science).

### Statistical methods

Statistical analyses were performed using SPSS software (version 15.0; SPSS, Chicago, IL, USA). Fisher's exact test was employed to determine the statistical significance of associations between cortactin, FADD, cyclin D_1_, and Pak1 protein expression, and *CCND1 *amplification. The Spearman's rank-order correlation coefficient (ρ), Kruskal-Wallis, and the Wilcoxon/Mann-Whitney tests were used for associations with clinicopathological parameters. To study RFS, the Kaplan-Meier method was used, and the log-rank test was applied for comparison of RFS survival among different treatment groups. A Cox proportional hazards regression model was used for the estimation of relative risk in univariate analysis. All *P *values corresponded to two-sided tests, and a *P *value less than 0.05 was considered statistically significant.

## Results

### The 11q genes *FADD *and *CTTN *are amplified at high frequencies, whereas *CHK1 *is deleted in primary breast tumors

**Figure 1:**
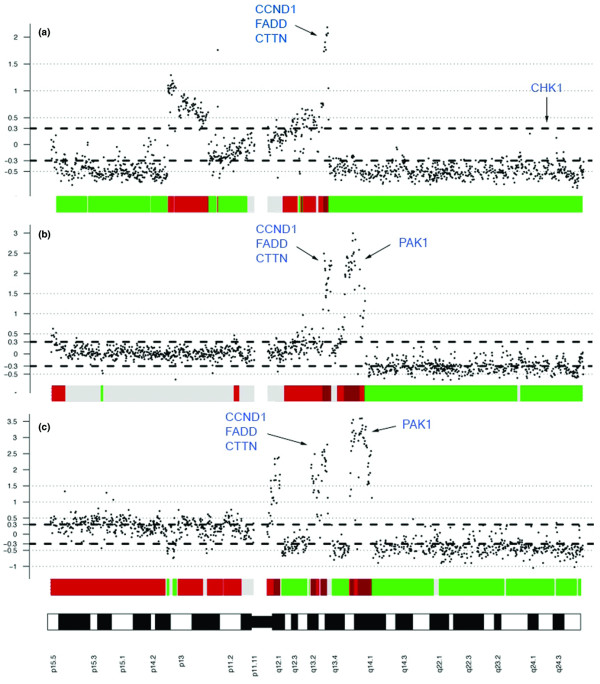
Genomic profiles of chromosome 11 from three breast tumors demonstrating different amplification patterns. Breast tumor samples were analyzed by array CGH. Bold dashed lines correspond to a log2(ratio) of ± 0.3 and represent gain or loss. The high-level peaks on 11q13 comprising (among others) *CCND1*, *FADD*, *CTTN *(panels a, b, and c) and *PAK1 *(panels b and c) represent gene amplification. The distal part of chromosome 11q, telomeric to the amplified region, including the *CHK1 *gene, has been hemizygously deleted, consistently among all three tumors. CGH, comparative genomic hybridization.

An array CGH analysis of more than 100 breast cancer samples (of which 56 were included in the clinical material) revealed a frequent gain of 11q13 genes, with concurrent deletion of distal 11q in many cases. *CCND1*, *FADD*, *CTTN*, and *PAK1 *were amplified at high frequencies, as illustrated by three representative samples in Figure 1. We therefore selected *FADD *and *CTTN *as candidate genes for further protein studies in addition to *CCND1 *and *PAK1 *(whose protein expression had previously been studied in the clinical material). Finally, the *CHK1 *gene was one of the potential cancer-related genes included in the deleted region and was thus selected to represent distal 11q deletion.

### Cortactin, FADD, and Chk1 are expressed in human breast cancer cell lines

**Figure 2:**
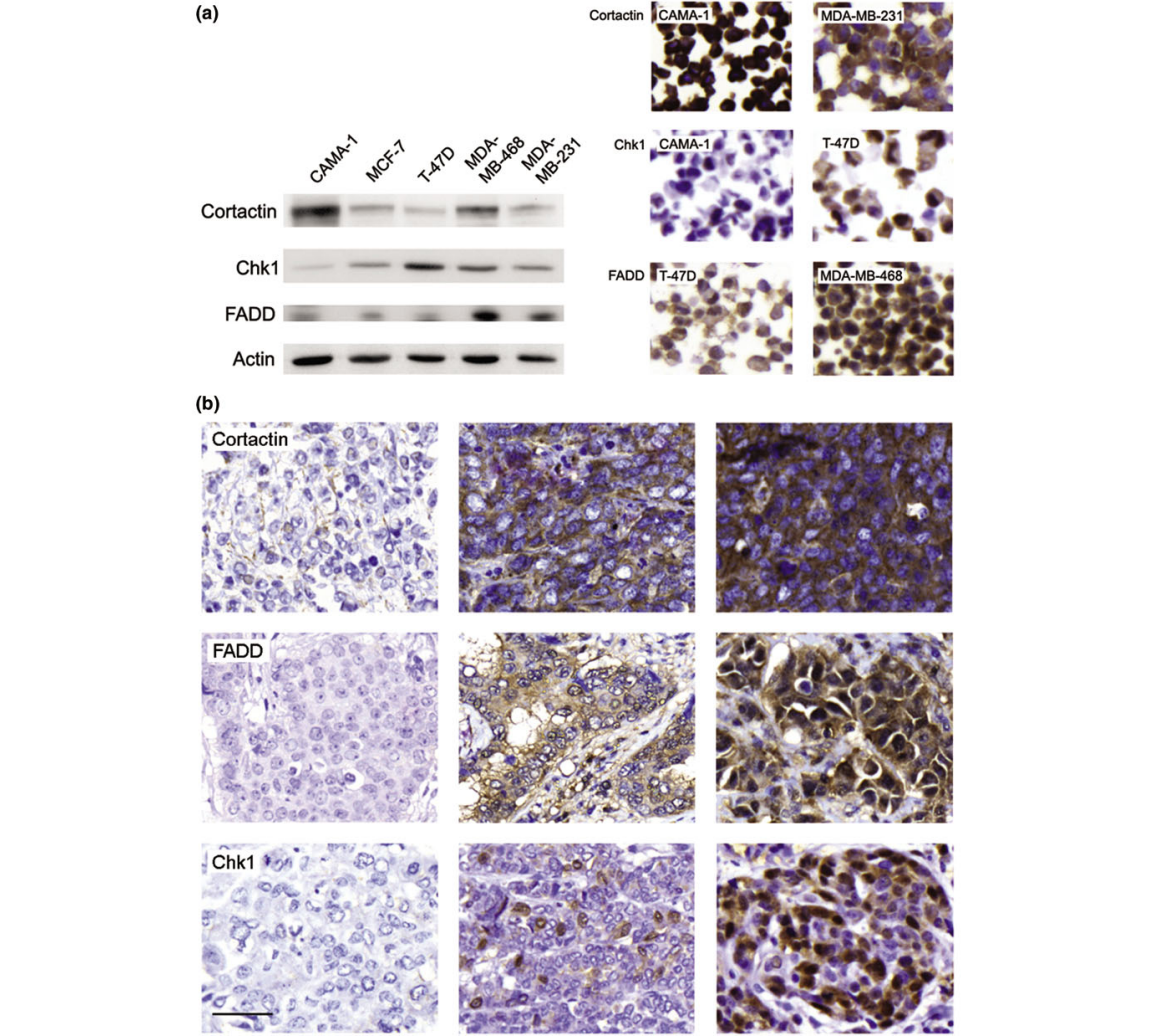
Expression of cortactin, FADD, and Chk1 in breast cancer cell lines and primary breast tumors. **(a) **Five human breast cancer cell lines were examined for cortactin, FADD, and Chk1 protein expression by Western blot and immunocytochemistry. Protein levels were equivalent between the two methods. **(b) **Tumor staining reveals cytoplasmic staining of cortactin and FADD, whereas Chk1 protein is mainly present in the nuclei. Staining intensity for cortactin and FADD was evaluated as negative (0) or low (1; left panels: cortactin low, FADD negative), intermediate (2; middle panels), and high (3; right panels). Chk1 staining was evaluated according to fraction positive nuclei (0% to 5% left panel, 6% to 50% middle panel, 51% to 100% right panel). In tumors with highly stained nuclei Chk1 was also present in the cytoplasm. Scale bar = 25 μm. Chk1, checkpoint kinase 1; FADD, Fas-associated death domain.

To validate the antibodies for detection of cortactin, FADD, and Chk1, their reactivity was tested in five different human breast cancer cell lines, by Western blot and ICC analyses. Bands corresponding to the predicted molecular weight for each protein were obtained at 80 to 85 kDa (cortactin), 23 kDa (FADD), and 54 kDa (Chk1; Figure 2a). The anti-FADD antibody also interacted with an additional unspecific protein product. The content of each protein observed by Western blot analysis corresponded well to the amount of protein detected by ICC in the same cell lines (Figure 2a), indicating (in accordance with a previous study [26]) that ICC was a valid method for quantification of the actual protein content.

### Cortactin and FADD are over-expressed in primary breast tumors

IHC staining was assessable in 258 of the 500 primary breast tumors for cortactin and in 295 for FADD. Based on the variation between tumor core staining intensity (observed in the initial examination of the cortactin and FADD stainings from test arrays), we defined four subcategories for cytoplasmic staining intensity: negative (0), low (1), intermediate (2), and high (3; Figure 2b). Tumors exhibiting high staining intensity (3) were classified as potentially over-expressing, with 70 out of 258 (27.1%) and 51 out of 295 (17.3%) of tumor samples over-expressing cortactin and FADD, respectively. Expression levels reported for cyclin D_1 _and Pak1 over-expression were 9.7% and 12.9%, respectively [17,26]. For statistical analysis, tumors were subcategorized as over-expressing or non-over-expressing, because the over-expressing fraction was the one possibly representing an amplification of the 11q13 locus.

### Expression of cortactin, FADD, cyclin D_1 _and Pak1 are positively correlated

Available data on cyclin D_1 _and Pak1 allowed for statistical testing of associations between expression of these two proteins, and cortactin and FADD. Only three tumors exhibited concurrent over-expression of cortactin, FADD, cyclin D_1_, and Pak1. Over-expression of cortactin, cyclin D_1_, and Pak1 was present in four out of 216 (1.9%) tumors; three out of 243 (1.2%) were FADD, cyclin D_1_, and Pak1 over-expressing; eight out of 212 (3.8%) over-expressed cortactin, FADD, and cyclin D_1_; and 10 out of 193 (5.2%) tumors over-expressed cortactin, FADD, and Pak1. Concurrent over-expression of only two proteins was somewhat higher, and significantly positive correlations between expression of any two of the four proteins were observed (cortactin/FADD [14.0%], *P *< 0.001; cortactin/cyclin D_1 _[4.5%], *P *= 0.049; cortactin/Pak1 [7.6%], *P *= 0.001; FADD/cyclin D_1 _[3.5%], *P *= 0.004; and FADD/Pak1 [6.0%], *P *< 0.001), but not between cyclin D_1 _and Pak1 (2.1%; *P *= 0.126). No link between expression of cortactin or FADD and tumor behavior, defined as tumor grade, type, size, lymph node status, and proliferation, was found. However, in the subgroup of tumors exhibiting ER-α positivity, FADD was positively correlated to proliferation (*P *= 0.002).

### Expression of the 11q13 gene products is associated with *CCND1 *amplification

Next, we examined the association between protein expression of cortactin, FADD, cyclin D_1_, and Pak1, and *CCND1 *amplification. In the same cohort amplification of *CCND1 *was observed in 15.7% of the tumors, by FISH analysis, as previously described by Jirstrom and coworkers [17]. *CCND1 *amplification was positively correlated to protein expression of cortactin (*P *= 0.020), FADD (*P *= 0.002), cyclin D_1 _(*P *< 0.001), and Pak1 (*P *= 0.008; Table 1). Of the *CCND1 *amplified tumors, 11 out of 25 (44.0%) exhibited over-expression of cortactin and 10 out of 24 (41.7%) over-expressed FADD.

**Table 1:** Fraction of tumors showing *CCND1 *amplification (FISH) in subgroups defined by expression of the 11q proteins

	Cortactin	FADD	Cyclin D_1_	Pak1	Chk1 fraction positive nuclei (%)			Chk1/Ki67 ratio	
						
					0 to 5	6 to 50	51 to 100	Deviant	Normal
*CCND1 *amplified (%)	14.6	12.6	15.9	16.3	24.2	15.3	0	34.8	14.8
Amplified and overexpessing (%)	44.0	41.7	31.8	28.9					
*P *value	0.020	0.002	<0.001	0.008	0.010			0.034	

*P *values for correlations between expression of the 11q gene products and *CCND1 *amplification are indicated in the bottom row. Chk1, checkpoint kinase 1; FADD, Fas-associated death domain; FISH, fluorescence *in situ *hybridization; Pak1, p21-activated kinase 1.

### Chk1 expression is associated with *CCND1 *amplification and an aggressive tumor phenotype

We next investigated potential associations between over-expression of the proteins with genes located at 11q13, and loss of Chk1, our marker for distal 11q deletion. The Chk1 staining was evaluated according to fraction positive nuclei from 0 to 4 (0 to 5, 6 to 10, 11 to 25, 26 to 50, and 51 to 100%; Figure 2b). Low Chk1 protein content was defined by 0 (≥5%) positive nuclei, and consequently 108 out of 341 (31.7%) tumors exhibited low levels of Chk1. The intermediate subgroup (6% to 50%) included 192 out of 341 (56.3%) tumors, and the high (51% to 100%) subgroup 41 out of 341 (12.0%) tumors. Over-expression of cortactin, FADD, cyclin D_1_, and Pak1 was not associated with loss of Chk1 protein expression. However, Chk1 expression inversely correlated to amplification of *CCND1 *(*P *= 0.010; Table 1), verifying a possible link between amplification of 11q13 and deletion of 11q24. Full sections of representative tumors exhibiting *CCND1 *amplification and low expression of Chk1 were stained using CISH to verify that tumor cells with low Chk1 expression actually were amplified in the *CCND1 *gene (data not shown).

Interestingly, the expression of Chk1 protein positively correlated to tumor grade (*P *< 0.001), tumor type (*P *= 0.001), and tumor size (*P *= 0.012), and in addition to expression of the proliferation marker Ki67 (*P *< 0.001; Table 2), suggesting that Chk1 is a marker for tumor aggressiveness.

**Table 2:** Distribution of Chk1 staining category according to clinico-pathological parameters

	Chk1 fraction positive nuclei (%)			
Variable	0 to 5 (*n* = 108)	6 to 50 (*n* = 192)	51 to 100 (*n* = 41)	*P *value
Tumor type				0.001^a^
Ductal	94	168	32	
Lobular	10	3	2	
Medullary	1	15	6	
Missing cases: 169				
Tumor size (mm)				0.012^b^
≥20	48	64	10	
>20	60	127	31	
Missing cases: 160				
Lymph node status				0.131^b^
Negative	24	54	14	
Positive	83	138	27	
Missing cases: 160				
NHG				<0.001^c^
I	18	14	0	
II	67	66	3	
III	20	106	36	
Missing cases: 170				
Ki67 positive (%)				<0.001^c^
0 to 10	70	55	2	
11 to 25	19	61	5	
26 to 100	10	52	32	
Missing cases: 194				

^a^Kruskal-Wallis test (two-sided). ^b^Mann-Whitney test (two-sided). ^c^Spearman's ρ. Chk1, checkpoint kinase 1; NHG, Nottingham Histological Grade.

In order to define further a subgroup of tumors exhibiting low Chk1 expression (with exclusion of false-negative Chk1 tumors that were Chk1 low as a consequence of low proliferation), we examined Ki67 expression in relation to Chk1. For a tumor to be included in this new subgroup termed 'Chk1 deviant', the expression of Chk1 (categories 0 to 4) had to be at least two staining categories lower than the expression of Ki67 (categories 0 to 4) in the same tumor. Consequently, this group included some tumors with a higher fraction of positive nuclei than the cutoff of 5% positive nuclei that defined the Chk1 low subgroup in previous analyses, and this subgroup included 45 out of 307 (14.7%) tumors. With this alternate classification of Chk1 protein expression, the only correlation observed was the inverse correlation with *CCND1 *amplification (*P *= 0.034; Table 1).

### *PAK1 *and *CCND1 *gene and protein expression are analogous in primary breast tumors

Of the 500 tumors available from our clinical material, 56 tumors had previously been analyzed by array CGH. These tumors included 48 cases from the untreated control arm and eight cases from the tamoxifen-treated arm. A log2(ratio) of ± 0.3 was used to represent gain or deletion, and values above 0.8 were considered amplification. The 56 tumors were categorized as deleted (under -0.3), normal (-0.3 to +0.3), or gained/amplified (over 0.3) in the *CCND1*, *FADD*, *CTTN*, *PAK1*, and *CHK1 *genes. Table 3 describes the associations between the protein expression and the gene expression for each of the 11q candidates. The limited number of tumor samples being analyzed for both CGH and IHC reduced the chances of obtaining a significant *P *value, but for *PAK1*/Pak1 a significant positive correlation was observed between protein and gene expression (*P *= 0.027; Table 3). For *CCND1*/cyclin D_1 _three of the four tumors over-expressing the protein were included in the gain/amplification subgroup, suggesting a link between protein over-expression and increased gene copy number, even though it did not reach statistical significance. No correlation between Chk1 deviant protein expression and *CHK1 *gene expression was found. Because none of the tumor samples analyzed by CGH were assessed as being *CCND1 *amplified by FISH, the *CCND1 *amplification status between these two methods could not be compared.

**Table 3:** Distribution of 11q protein staining category in relation to CGH gene profile

Protein expression	Gene expression				
	Deletion	Normal	Gain/Amp	Total	*P *value^a^
Cortactin					0.664
Low/intermediate expression	1	13	5	19	
Over-expression	0	6	3	9	
Total	1	19	8	28	

FADD					0.477
Low/intermediate expression	0	16	7	23	
Over-expression	0	3	3	6	
Total	0	19	10	29	

Cyclin D_1_					0.083
Low/intermediate expression	3	30	10	43	
Over-expression	0	1	3	4	
Total	3	31	13	47	

Pak1					0.027
Negative	3	28	4	35	
Positive	0	4	5	9	
Total	3	32	9	44	

Chk1					0.241^b^
0% to 5% (low)	2	6	0	8	
6% to 50% (intermediate)	5	18	0	23	
51% to 100% (high)	0	7	0	7	
Total	7	31	0	38	

^a^Correlations were calculated using Mann-Whitney test (two-sided) unless otherwise specified. ^b^Spearman's ρ. Amp, amplification; CGH, comparative genomic hybridization; Chk1, checkpoint kinase 1; FADD, Fas-associated death domain; Pak1, p21-activated kinase 1.

### Deviant expression of Chk1 is associated with impaired response to tamoxifen

**Figure 3:**
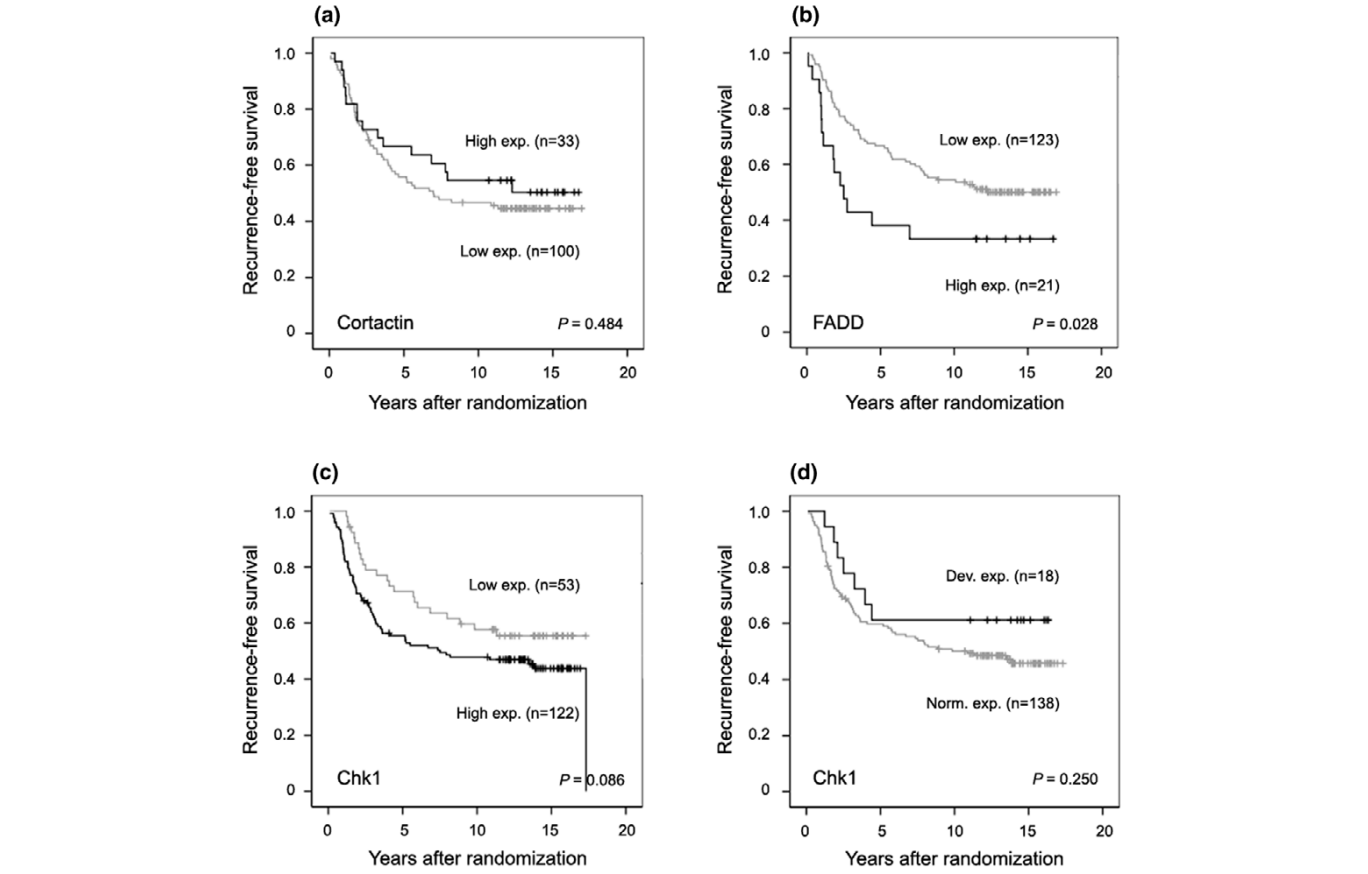
Recurrence-free survival according to protein expression of cortactin, FADD, and Chk1. Kaplan-Meier curves showing the effect of **(a) **cortactin, **(b) **FADD, **(c,d) **and Chk1 expression on recurrence-free survival in the subgroup of untreated control patients. Expression of cortactin or Chk1 did not influence the recurrence rate. However, over-expression of FADD was associated with shorter recurrence-free survival compared with lower expression. Chk1, checkpoint kinase 1; FADD, Fas-associated death domain.

The tumor material collected from a clinical trial of patients randomly assigned to either tamoxifen or no adjuvant treatment after surgery gave us the unique opportunity to study survival in the subgroup of patients not receiving adjuvant therapy. The expression of cortactin (Figure 3a) and Chk1 (Figure 3c,d) was not associated with any effect on RFS (cortactin, *P *= 0.484; Chk1, *P *= 0.086; and Chk1 [normal/deviant], *P *= 0.250) in this subgroup of patients. However, over-expression of FADD was associated with a shorter RFS, as compared with lower expression (*P *= 0.028; Figure 3b). A multivariate Cox regression analysis nevertheless revealed no independent prognostic value for FADD expression (data not shown).

**Figure 4:**
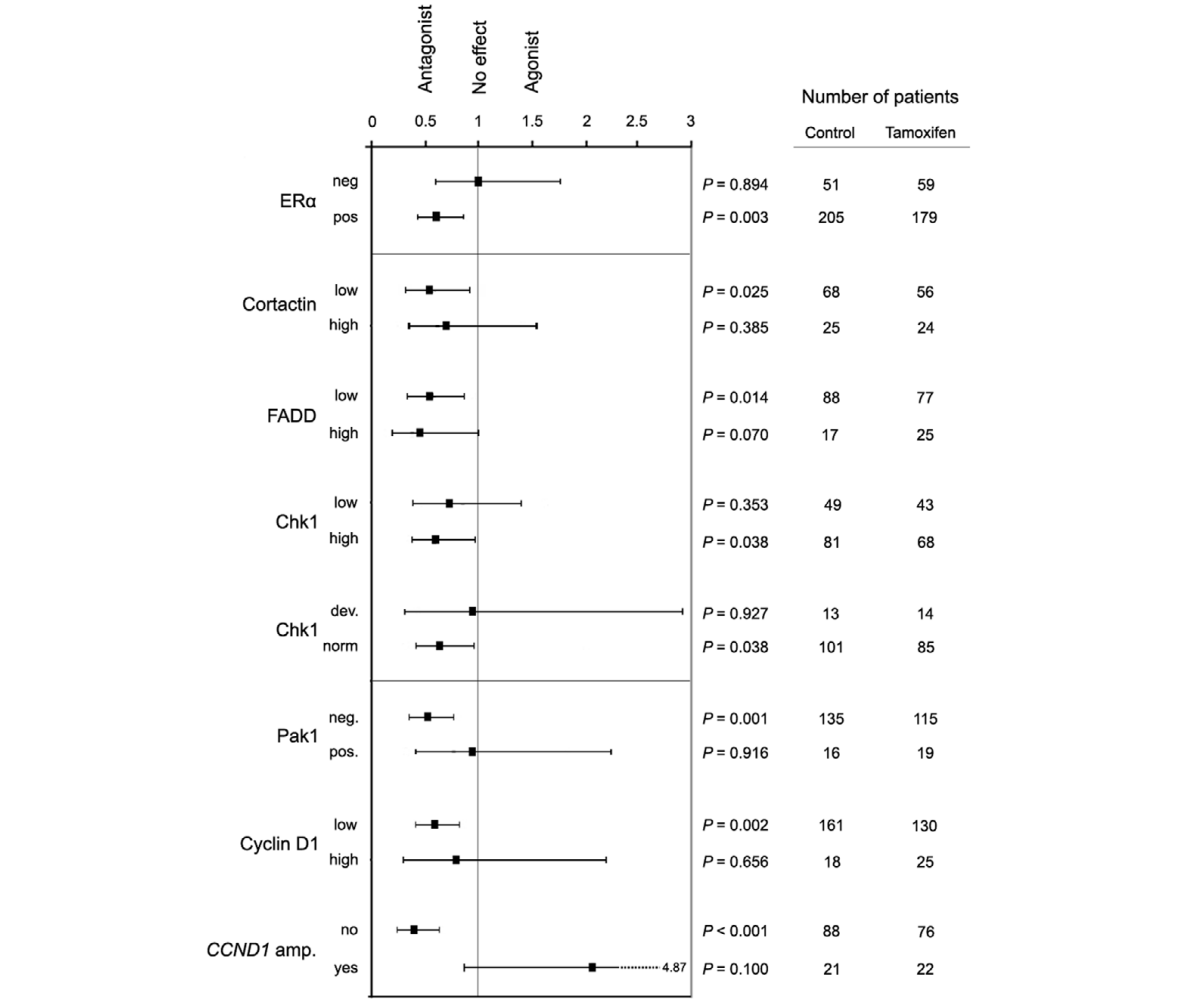
Predicted tamoxifen response in patient subgroups defined by protein expression of the 11q gene products. Hazard ratios (black boxes, with 95% confidence intervals) were calculated for each subgroup in univariate analysis using a Cox proportional hazards regression model. The number of patients in each subgroup receiving no adjuvant treatment versus tamoxifen, and *P *values are indicated. In patients with ER-α-positive tumors tamoxifen had an antagonistic effect, whereas patients with ER-α-negative tumors did not respond to tamoxifen. The expression of cortactin and FADD did not influence the tamoxifen response significantly, whereas patients with tumors showing deviant Chk1 expression did not respond to tamoxifen, relative to patients with tumors exhibiting normal expression. Tamoxifen response was impaired in patients with tumors exhibiting over-expression of Pak1 (defined as positive nuclei) or cyclin D_1_, and the effect was potentially agonistic in patients with *CCND1 *amplified tumors. Chk1, checkpoint kinase 1; ER, estrogen receptor; FADD, Fas-associated death domain; Pak1, p21-activated kinase 1.

Next, the expression of cortactin, FADD, and Chk1 was analyzed in association with tamoxifen response in patients with ER-α-positive tumors. These patients were selected on the basis that they would be the ones most likely to respond to an ER-α-targeted therapy. The predicted response to tamoxifen in different subgroups defined by cortactin, FADD, and Chk1 expression was considered in a univariate Cox regression analysis (Figure 4). The tamoxifen response in the different subgroups was compared with the response of both patients with ER-α-positive tumors, who benefited from 2 years of adjuvant therapy with tamoxifen [25], and patients with ER-α-negative tumors who, in contrast, did not benefit. Moreover, the effect of tamoxifen in subgroups defined by cyclin D_1 _and Pak1 protein expression and amplification of *CCND1 *is presented in Figure 4.

**Figure 5:**
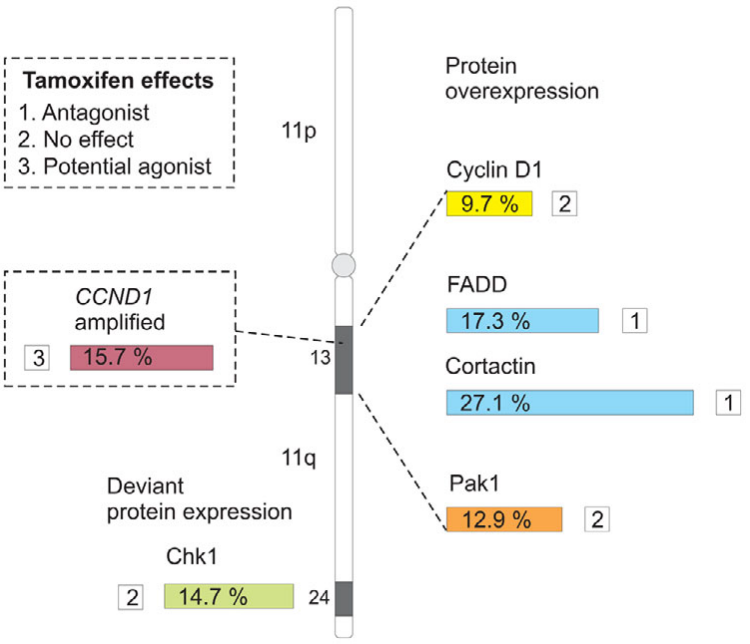
11q protein data in the cohort of premenopausal breast cancer patients. Protein expression of the 11q gene products and the associated response to tamoxifen was examined in primary breast tumors. Chk1, checkpoint kinase 1; FADD, Fas-associated death domain; Pak1, p21-activated kinase 1.

Interestingly, when comparing patients who received tamoxifen versus those receiving no adjuvant treatment, the expression of Chk1 (defined as deviant or normal expression) appeared to be of importance for the response, whereas cortactin and FADD was not. However, a trend indicating a slightly decreased tamoxifen response in patients with tumors exhibiting low Chk1 or high cortactin expression was observed. The effect of tamoxifen was clearly impaired in patients with tumors showing deviant Chk1 expression (*P *= 0.927), whereas a positive effect of tamoxifen was identified in the subgroup representing a more typical Chk1/Ki67 expression ratio (*P *= 0.038). In an attempt to assess whether the effect of tamoxifen treatment on RFS differed between subgroups as defined by Chk1 expression being normal or deviant, a Cox model with main effects for Chk1 and tamoxifen treatment and interaction term (Chk1 treatment) was tested. However, this analysis did not identify a significant difference in tamoxifen treatment effect on RFS between the normal and the deviant subgroups (*P *= 0.374; data not shown). Figure 5 summarizes the fraction of tumors over-expressing cortactin, FADD, cyclin D_1_, and Pak1, the fraction exhibiting deviant Chk1 expression, and the responsiveness to tamoxifen in tumors showing altered expression of any of these proteins. In patients with tumors exhibiting *CCND1 *amplification, the effect of tamoxifen was potentially agonistic instead of antagonistic.

## Discussion

The amplification event at chromosome locus 11q13 has in a number of different cancers been associated with unfavorable prognosis [6,7]. Several genes included in this region have been identified as driver genes, or most frequently amplified genes [3,7,18]. These driver genes are putative promoters of biologic processes such as oncogenesis and multidrug resistance [18,29].

*CTTN *has been proposed to be a strong candidate gene driving 11q13 amplification [7], and clear evidence for involvement of the F-actin binding gene product cortactin in tumorigenesis have been reported [1,3,30]. FADD has been implicated in cell survival as well as growth control, and consequently it may play a role in tumor progression [31]. Furthermore, *FADD *has been reported to be a candidate driver gene for the 11q13 amplification in HNSCC [18].

In this study, the search for candidate genes co-amplified with *CCND1 *and responsible for impaired tamoxifen response was conducted by assessing the expression of two proteins with corresponding genes at the 11q13 locus, and one gene harbored at the distal chromosome 11q, namely *CHK1*. Importantly, the protein expression should not be seen as an exact reflection of the amplification event, but a certain conformity of amplification and expression level is expected. Protein over-expression is not exclusively caused by amplification, but it can be the result of several different genetic alterations. The co-variance between the expression of cortactin, FADD, cyclin D_1_, and Pak1 might be interpreted as a reflection of the amplification event. Over-expression of more than one of the four 11q13 proteins was highest in subgroups expressing high levels of both cortactin and FADD, and might be explained by the chromosomal location, where the *CTTN *and *FADD *genes are located in close proximity. The concurrent protein over-expression with the lowest overlay was between cyclin D_1 _and Pak1, which may be due to the location of these two genes on either side of region harboring *CTTN *and *FADD*. The positive correlation between expression of cortactin, FADD, cyclin D_1_, and Pak1 further suggests a possible link to co-amplification of the different core regions at the 11q13 locus. However, no correlation between cyclin D_1 _and Pak1 was observed, and this further confirms that these two genes may not be co-amplified to the same extent as genes in closer proximity.

In contrast, *CCND1 *amplification was positively correlated with expression of all four proteins, indicating a link between amplification of the four genes. Of the *CCND1 *amplified tumors, between 28.9% and 44.0% over-expressed one of the four proteins, indicating that amplification of *CCND1 *might be of importance for amplification of any of the other three genes we examined at the 11q13 locus. The fraction of Pak1 over-expressing tumors was (in the *CCND1 *amplified subgroup) again the lowest of the four 11q13 proteins, indicating a lower frequency of co-amplification of *CCND1 *and *PAK1*.

Chk1 is one of the key regulatory components of the DNA damage checkpoint and its corresponding gene has been mapped to 11q24, and was used as our marker for the deletion occurring at distal 11q. The inverse correlation between Chk1 protein expression and *CCND1 *amplification might be interpreted such that *CCND1 *amplification also involves a deletion of distal chromosome 11q, and in this case a loss of heterozygosity (LOH) of the *CHK1 *gene, resulting in a lower level of protein expression. Mapping of *CHK1 *to this chromosomal region of frequent LOH in human tumors indicates that this gene is a putative tumor suppressor gene [32]. The positive correlation between Chk1 protein expression and tumor grade, tumor type, tumor size, and Ki67 expression defines Chk1 as a marker for tumor aggressiveness. The subgroup exhibiting low Chk1 expression could hypothetically have a defective DNA damage response, resulting in a more aggressive tumor. However, when analyzing prognostic features of Chk1 in untreated premenopausal breast cancer patients, we did not observe any link between Chk1 and increased recurrence rate.

The definition of Chk1 expression in relation to proliferation characterized a subgroup of Chk1 low-expressing tumors exhibiting a high proliferation rate, excluding false-negative tumors. Within this subgroup, only the inverse correlation with amplification of *CCND1 *was observed, suggesting an accurate classification and a better representation of *CHK1 *deleted tumors.

This tumor material has been used in several different studies thus far, and extensive sectioning has led to a significant number of missing tumor cores. When analyzing the tumors that were missing for the 11q genes (102 out of 514), there was a tendency toward over-representation of lobular cancers and cancers of low grade and low proliferation, but there was no difference in breast cancer recurrences.

Because the overlay between CGH and the IHC analyses consisted of only 56 tumors, the statistical power of these analyses was limited. However, the significant correlation between the gene and protein expression of *PAK1*/Pak1 indicates that an association between the expression of the other 11q genes and their protein products is also likely. As previously described, the correlation between gene and protein expression was confirmed for *CCND1*/cyclin D_1 _with the FISH data available. No correlation between gene and protein expression for *CHK1*/Chk1 was found. Using the definition of Chk1 as normal or deviant did not reveal any further information about the association between the CGH and IHC data.

Tamoxifen resistance is commonly encountered in breast cancer therapy, with approximately one-third of ER-α-positive breast cancers resistant to the drug. Hence, clarification of the underlying cause of resistance could prove vital in augmenting treatment strategies. Because we previously found amplification of *CCND1 *to be associated with an adverse effect of tamoxifen in premenopausal patients, it may be expected that over-expression of cyclin D_1 _protein would show the same trend. During the amplification event at chromosome locus 11q13, more than one region can be amplified, meaning *CCND1 *may be expressed in addition to several other genes. It is entirely plausible that one such gene may be responsible for the adverse effect of tamoxifen.

In this cohort of randomized premenopausal breast cancer patients, we observed that – with high expression of cortactin and low expression of Chk1 – there was a tendency toward an impaired response to tamoxifen. Interestingly, patients with tumors exhibiting Chk1 expression defined as deviant clearly did not have an improved RFS when treated with tamoxifen.

Because over-expression of either cyclin D_1 _or Pak1 impaired the tamoxifen response in the patient cohort, it was of interest to investigate the influence of combined over-expression of cyclin D_1 _and Pak1 on the outcome of tamoxifen response. However, only eight patients with ER-α-positive breast cancer exhibited combined over-expression, limiting the statistical power of this analysis. In clinical material including a larger number of patients, this type of analysis might reveal pertinent information regarding combined over-expression of cyclin D_1 _and Pak1, and also for cyclin D_1 _over-expression in combination with deviant Chk1 expression.

The underlying mechanism for tamoxifen resistance in subgroups of tumors exhibiting deviant Chk1 expression is unclear. In the case of cyclin D_1 _and Pak1, the resistance mechanism could be explained by both cyclin D_1 _and Pak1 potentiating the activity of the ER-α [33-35]. Notably, Chk1 was used in this study as a marker for distal 11q deletion, but we can not state whether loss of *CHK1 *alone or deletion of a larger chromosomal region of 11q is the important event in breast cancer that leads to decreased tamoxifen response. Nevertheless, there is a multitude of gene products that are potentially downregulated in conjunction with a loss of the distal end of 11q, and it is unlikely that loss of *CHK1 *is the only important event on 11q.

Climent and coworkers [36] reported that deletion of 11q in node-negative breast cancer was associated with earlier relapse in patients not receiving anthracycline-based chemotherapy, as compared with patients receiving this kind of treatment. The distal part of chromosome 11q harbors a number of genes that are involved in DNA repair, and hence a deletion of this region might contribute to defective repair machinery. Two major genes that are involved in DNA repair and cell cycle control are the ataxia-telangiectasia mutated (*ATM*) gene and the *CHK1 *gene [37,38]. These two genes, together with a number of additional ones involved in DNA repair, have been proposed as candidate targets for the 11q deletion [39,40].

Several genetic alterations that differed between the tamoxifen-sensitive breast cancer cell line MCF-7 and the tamoxifen-resistant clone CL-9 were identified in a study based on CGH analyses [41]. One of the alterations that was seen exclusively in the tamoxifen-resistant cell line was the deletion of 11q24, indicating that genes that are involved in development of tamoxifen resistance are potentially harbored in this chromosomal region. The mechanism of endocrine responsiveness in breast cancer is thought to be controlled by complex interactions between steroid hormones and numerous signaling pathways, such as growth factor signaling, which in turn most likely can be affected by different genetic alterations [42]. The identification of genes responsible for, or involved in, tamoxifen resistance is one approach to clarify the underlying mechanisms in this intricate series of events. To date, few reports have dealt with genetic alterations and anti-estrogen resistance; it is therefore of great importance to continue the search for possible markers involved in this elusive field of breast cancer biology.

Presence of the progesterone receptor (PR) is considered to be an indicator of a functional ER, and thus determines the extent of the response to hormonal therapy [43]. The PR gene maps to chromosomal region 11q22–23 [44] and is consequently likely to be involved in the LOH occurring at distal 11q, where the whole end telomeric to 11q14 commonly is lost. In a previous report by Stendahl and coworkers [45] it was observed that expression of the PR was a stronger predictor of tamoxifen response than the ER. This indicates that the mechanism responsible for loss of this receptor gene is important and, although CGH analyses for this particular genetic event is beyond the scope of the present study, it suggests a productive area for future research. Interestingly, we observed an association between expression of PR and Chk1 defined as normal or deviant, with borderline significance (*P *= 0.054; data not shown).

When considering candidate genes responsible for altered disease outcome in different cancers related to 11q13 amplification, the deletion of distal 11q should clearly be considered an event that might be equally important for disease progression and tamoxifen response. Even though a number of genes involved in tumorigenesis have been proposed so far, further studies investigating LOH of genes at distal 11q would be needed to characterize the main candidates with roles in tumor behavior and treatment response. The biological mechanism that interconnects the protein expression of Chk1 and reduced tamoxifen sensitivity needs to be explored. It is apparent that future studies are necessary to determine the intricate mechanisms underlying tamoxifen resistance, and to elucidate the cause of events rendering *CCND1 *amplified premenopausal breast cancers not only resistant but possibly stimulated by this selective ER modulator.

## Conclusion

By CGH analysis of breast cancer samples, we identified *CTTN *and *FADD *as co-amplified with *CCND1 *at the 11q13 locus, and *CHK1 *as a marker for the frequently occurring distal 11q deletion. When analyzing expression of the associated gene products by immunohistochemistry in tissue specimens from premenopausal breast cancer patients randomized to either tamoxifen or no adjuvant treatment, we observed that over-expression of cortactin and FADD as well as downregulation of Chk1 was linked to *CCND1 *amplification. Furthermore, deviant Chk1 expression was associated with an impaired tamoxifen response. However, none of the 11q gene products was linked to an agonistic effect of tamoxifen, as reported for *CCND1 *amplified tumors. Our findings demonstrate that 11q deletions may be involved in tamoxifen resistance in breast cancer.

## Abbreviations

CISH: chromogenic *in situ *hybridization; ER: estrogen receptor; CGH: comparative genomic hybridization; Chk1: checkpoint kinase 1; FADD: Fas-associated death domain; FISH: fluorescence *in situ *hybridization; HNSCC: head and neck squamous cell carcinoma; ICC: immunocytochemistry; IHC: immunohistochemical; LOH: loss of heterozygosity; PR: progesterone receptor; RFS: recurrence-free survival.

## Competing interests

The authors declare that they have no competing interests.

## Authors' contributions

KL carried out the IHC assessments, performed the statistical analyses and drafted the manuscript. KH performed the CGH analyses, interpreted the data from these analyses, and revised the manuscript. BN contributed to the planning and performance of the clinical trial and revised the manuscript critically. ÅB supervised the CGH study design and revised the manuscript. GL participated in the study design and interpretation of the data, took part in the IHC assessments, and helped to draft the manuscript. All authors read and approved the final manuscript.
